# Lack of EGFR mutations benefiting gefitinib treatment in adenocarcinoma of esophagogastric junction

**DOI:** 10.1186/1477-7819-10-14

**Published:** 2012-01-17

**Authors:** Wen-Ping Wang, Kang-Ning Wang, Qiang Gao, Long-Qi Chen

**Affiliations:** 1 Department of Thoracic Surgery, West China Hospital, Sichuan University, Chengdu 610041, China

**Keywords:** Epidermal growth factor receptor, Adenocarcinoma of esophagogastric junction, Gene mutation, Single nucleotide polymorphism, Gefitinib

## Abstract

**Background:**

The epidermal growth factor receptor (EGFR) inhibitor, gefitinib, has been reported to successfully treat advanced non-small cell lung cancer patients with genetic mutations in EGFR. The aim of this study was to investigate the existence of EGFR mutations in carcinoma of esophagogastric junction, and also to explore the possibility of treating carcinoma of esophagogastric junction using gefitinib.

**Methods:**

From Aug. 2009 to Jun. 2010, 65 patients with carcinoma of esophagogastric junction underwent surgical resection. The tumor tissue and corresponding blood specimens were collected from all cases. The DNA was extracted and PCR amplification was accomplished based on designed primers for exons 18, 19, 20, and 21. EGFR exons 18, 19, 20 and 21 of both cancer cell and white blood cell were finally successfully sequenced.

**Results:**

In exon 20, a variant from CAG to CAA at codon 787 (2361G-> A) was identified in 19 patients, which was a genomic variation of EGFR since it was found in both cancer tissue and white blood cells. This EGFR alteration was a synonymous single nucleotide polymorphism (SNP) since CAA and CAG were encoding the same amino-acid of Glutamine (Q787Q, NCBI database 162093G > A, SNP ID: rs10251977). No genetic alteration was found in exons 18, 19 or 21.

**Conclusions:**

Adenocarcinoma of esophagogastric junction rarely presents EGFR mutation, especially gefitinib-associated mutations such as L858R, or delE746-A750. This means that the gefitinib-based gene target therapy should not be recommended for treating carcinoma of esophagogastric junction.

## Background

Epidermal growth factor receptor (EGFR) plays an important role in the proliferation, apoptosis regulation, and protein secretion of the cells [1]. Human EGFR gene locates at chromosome 7p11-13 including 28 exons, which transduces and synthesizes a 179 KD Tyrosine Kinase (TK) family membrane protein consisting of 1186 amino acids. EGFR is structurally composed of three parts as extracellular ligand binding area, transmembrane (TM) and intracellular endogenous TK activity domain. After binding of EGFR and ligands such as EGF or TGF-α, the intracellular TK domain is activated and subsequent downstream biological signal transduction is stimulated resulting in the regulation of cell proliferation [2]. EGFR overexpression is currently discovered in some solid tumors of lung, breast, prostate, colon, ovary, gastrointestinal tract, head and neck. It can be the target of cancer therapy using small molecule inhibitors like special EGFR-TK targeted inhibitor to treat EGFR-overexpression tumors [3].

The EGFR-TK specific small molecule inhibitor (TKI) of Iressa (Gefitinib) was approved by FDA (U.S Food and Drug Administration) for advanced non-small cell lung cancer (NSCLC) treatment in May 2003 [4]. But the application of gefitinib suggested that just 10%-15% of the patients presented significant response [5]. Further studies revealed that just the tumor cell with EGFR-TK mutation (L858R, del742-759) could match good response to gefitinib [6,7].

According to the previous researches on EGFR DNA sequencing in Japan, South Korea and China, the incidence of EGFR mutation of NSCLC in Asian population was significantly higher than that in Europe or America. Also it was more frequent in female or non-smoking or adenocarcinoma patients. Recently a new EGFR mutation of T790M was discovered that explained the resistance to gefitinib after drug administration [8]. On the other hand, based on the mutation mechanisms above pharmacologist could design new inhibitors targeting EGFR mutation locus. In esophageal squamous cell carcinoma (ESCC), about 30.8% of the tumor cells presented EGFR overexpression in relation to the bad prognosis and long-term survival. This pointed out the possibility of TKI application in ESCC treatment. Phase I and II clinical trials of gefitinib in ESCC treatment were being carried out [9,10].

Carcinomas of the esophagus and esophagogastric junction (EGJ) are both common malignancies in China. For example, the annual incidence of carcinoma of EGJ was as high as 17.25/100 000 from 1988 to 2002 in the high incidence area of Ci County in northern China and currently the incidence was still rising yearly [11,12]. It locates at esophagogastric junction but may differ from either esophageal carcinoma or gastric cancer pathologically and clinically. Majority of the patients with dysphagia symptom are diagnosed as advanced stage so that the surgical treatment is not effective and satisfactory. It has been reported that the resectability of carcinoma of esophagogastric junction was 84.6% and 5-year survival rate was as low as 20.9% [13]. Therefore the postoperative adjuvant chemotherapy is usually recommended.

No study on EGFR mutation in adenocarcinoma of esophagogastric junction has been reported so far as we known. Preliminary immunohistochemical analysis discovered that more than 32% of the patients with esophagogastric junction carcinoma presented EGFR overexpression, which related with tumor stage, lymph node metastasis and postoperative tumor-free survival.

In this study, we planned to extract DNA from cancer tissue and corresponding peripheral blood cell from patients with EGJ cancer, then perform DNA sequencing of EGFR-TK domain including exons 18, 19, 20 and 21 in order to assess the EGFR mutation in carcinoma cell initially and predict the therapeutic effect of small molecule inhibitor of gefitinib on EGJ cancer.

## Methods

From Aug. 2009 to Jun. 2010, 65 patients with carcinoma of esophagogastric junction underwent surgery in our department (Table 1). The cancer tissue and corresponding peripheral blood specimen were obtained from these patients and stored temporarily in -80°C freezer. There were 61 men and 4 women. Twenty-one patients declared the smoking history (all were men, 18 were current smokers and 3 were ex-smokers), with the mean smoking index of 280 cigarettes per year (range: 120-500 cigarettes per year). All the patients were diagnosed as adenocarcinoma and had no any preoperative neoadjuvant therapy.

**Table 1:** Patients characteristics

	**No**.
Patients	65
Mean age(years)	64(range, 41-76)
Sex(male/female)	
Male	61
Female	4
Smoking history	21
Histological type	
Well differentiated	21
Moderately differentiated	28
Poorly differentiated	16

DNA of the tumor tissue and white blood cells was successfully extracted by routine proteinase K digestion and precipitated with TIANamp Blood/cell/tissue genomic DNA extraction kit (Tiangen, Beijing, China) according to the manufacturer's instruction.

In order to amplify EGFR exons 18, 19, 20 and 21 that located in EGFR-TK domain, we designed the PCR primers by using Primer Premier 6.0 (Premier, Canada). The designed primers were checked repeatedly and of which the specificity were also confirmed at website of In-silico PCR (http://genome.ucsc.edu/cgi-bin/hgPcr?command=start). Exon 18: Forward: 5'-CAA GTG CCG TGT CCT GG-3'; Reverse: 5'-AAA TGC CTT TGG TCT GTG AA-3', Exon 19: Forward: 5'-ATA TCA GCC TTA GGT GCG G-3', Reverse: 5'-GGG AAA GAC ATA GAA AGT GAA CA-3', Exon 20: Forward: 5'-TTC ACA GCC CTG CGT AAA C-3', Reverse: 5'-TTG AAT CCA AAA TAA AGG AAT GT-3'; Exon 21: Forward: 5'-TGG TCA GCA GCG GGT TAC-3', Reverse: 5'-TCA TTC ACT GTC CCA GCA AG-3'. PCR amplification was carried out on ABI 9700 PCR thermal cycler (Applied Biosystems, USA) in a 20 uL reaction system containing 10 ul of SUPER Taq Mix 2× (dNTP, Mg^2+ ^and Buffer), 7 ul of ddH_2_O, 1 ul of forward (F) primer, 1 ul of reverse (R) primer and 1 ul of DNA template. The PCR cycling conditions consisted of an initial denaturation step at 94°C for 2 min, followed by 35 cycles of 94°C for 20 s, 52°C for 10 s, 72°C for 30 s, and final extension step at 72°C for 2 min. The reactions were then held at 4°C before analysis.

The PCR products were purified with PCR product purification kit (BioDev-Tech, Beijing, China) according to manufacturer's instructions and sequenced directly using the Applied Biosystems sequencer 9700 according to the manufacturer's instruction. All sequence variants were confirmed by sequencing the products of independent PCR amplifications in both directions.

## Results and Discussion

**Figure 1:**
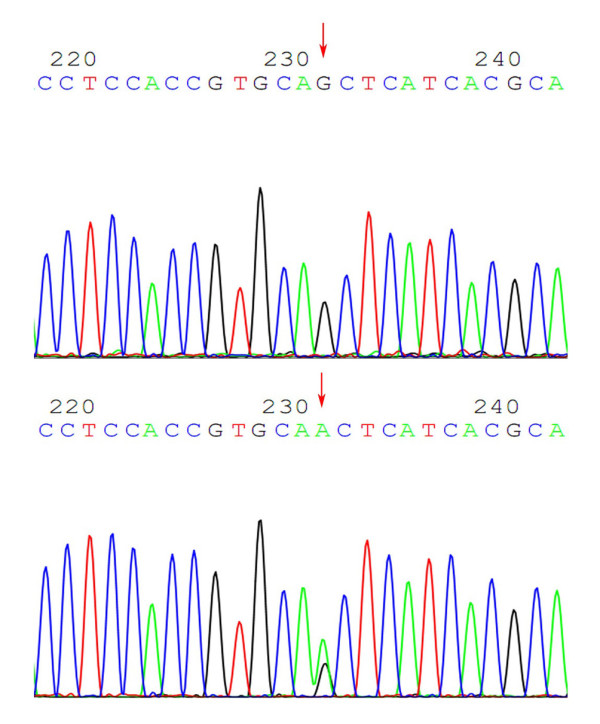
**The variant from CAG to CAA at codon 787 in exon 20**. The variant from CAG to CAA at codon 787 in exon 20 (arrows), which was a genomic variation of EGFR since it was found in both cancer tissue and white blood cells, and this was a synonymous mutation because CAA and CAG were encoding the same amino-acid of Glutamine (Q787Q).

The DNA sequencing of exons 18, 19, 20 and 21 was successfully performed and output with the *. ABL format file by Chromas 2.0. We analyzed all the DNA sequences. In exon 20, a variant from CAG to CAA at codon 787 (2361G-> A) was identified in 19 patients (29.2%) (Figure 1), which was a genomic variation of EGFR since it was found in both cancer tissue and white blood cells. No other variants were discovered in exons 18, 19, or 21. The discovered EGFR variant in exon 20 was a synonymous mutation because CAA and CAG were encoding the same amino-acid of Glutamine (Q787Q). It was considered as single nucleotide polymorphism (SNP). This SNP has been deposited in NCBI database (162093G > A, SNP ID: rs10251977).

The translated amino acids from DNA form the primary structure of protein. Furthermore, the steric configuration of the protein is functional structure which is preserved by molecular linkage between amino acids. If some particular amino acid is substituted, the molecular linkage should be changed resulting in the protein function alteration. Take L858R as example, which is a frequent EGFR mutation in NSCLC where leucine (L) is substituted by arginine (R) at the locus. The chemical distance between these two amino acids is 102 (Grantham, 1974), so the steric configuration of EGFR in this domain would be changed and this structural alteration facilitates the combination between TKI and EGFR-TK. This hypothesis may explain the sensitivity to gefitinib of the patients with EGFR L858R mutation. From this concern, if similar mutation exists in carcinoma of esophagogastric junction, the TKI therapy to carcinoma of esophagogastric junction will be promising.

In our study, carcinoma of esophagogastric junction rarely presents EGFR mutations, especially gefitinib-associated mutations such as L858R and delE746-A750. This is different from NSCLC, gastric cancer, esophageal carcinoma, and colonic or pancreatic carcinoma. This suggests the gefitinib-based small molecular target therapy is not appropriately applied in treating EGJ cancer. A similar result was found in study of Barrett's adenocarcinoma [14]. In exons 19 and 21 of EGFR, just K754K was found without other mutation identified. It was concluded that mutations within the tyrosine kinase domain of EGFR associated with sensitivity of NSCLC patients to gefitinib were not present in Barrett's adenocarcinoma. Some study revealed that the kinase domain of EGFR was highly conserved in whole gastric cancer cell lines and cases, therefore treatment with gefitinib should not be recommended for such malignancy [15]. A meta-analysis showed that the incidence of EGFR mutations in NSCLC varied according to cigarette-smoking history [16]. The detected EGFR mutations in exons 18, 19, and 21 of the patients with lung cancer were 48.6% in never smokers, 33.9% in former smokers, and 16.6% in current smokers. The mutations were less common in people who smoked for more than 15 pack-years or who stopped smoking cigarettes less than 15 years ago [17]. The presence of EGFR exon 19 deletions and L858R in patients with lung adenocarcinoma were found in 15% from former smokers, 6% from current smokers and 52% from never smokers [18]. However, there was little report on the correlation between smoking history and EGFR mutation in gastrointestinal carcinoma. In our study, all 65 patients with adenocarcinoma of EGJ, of which 21 cases were current or former smokers, presented none EGFR sensitive mutation. The correlation between smoking history and EGFR mutation in adenocarcinoma of EGJ was not definitely clear here and needs further investigation.

Besides the lack of meaningful EGFR mutation, we found that a synonymous SNP in exon 20 of EGFR gene. SNP is the DNA sequence variation caused by the single nucleotide alteration which exists widespread in human genome with the frequency more than 1%. SNPs may locate within coding sequences of genes, non-coding regions of genes, or in the intergenic regions. SNPs within a coding sequence may not change the amino acid sequence of the translated protein, due to degeneracy of the genetic code. A SNP in which both forms lead to the same polypeptide sequence is termed synonymous, as is the case of Q787Q in our study.

Previous studies on EGF gene were carried out and concluded that the SNP of EGF +61 G/A polymorphism was associated with ESCC in a Chinese population and the variant genotypes of GA/AA were associated with a significantly decreased risk of ESCC compared with the wild-type homozygote GG [19]. Other investigation suggested no association between EGF gene polymorphism and gastric cancer [20].

Choi and co-workers screened several EGFR SNPs in lung cancer cell and five SNPs were identified including 127378C > T, 142285G > A, 162093G > A, 181946C > T and 187114T > C. Meanwhile the SNP of 181946C > T was found be associated with the incidence of lung cancer [21]. So the SNP could be used as predictive marker for the genetic susceptibility to lung cancer according to their study. Kaneko et al reported the same SNP discovery of Q787Q with the rate of 33% in the esophageal squamous cell carcinoma cell, and a significant difference was seen in the overall survival between patients with and without the EGFR heterozygous genotype in their study [22]. Could this SNP of Q787Q be a clinically useful biomarker for predicting the prognosis of ESCC or EGJ cancer patients? It will be a promising study and need much further research. Also more studies are warranted to identify the relationship between this SNP change and the biological features and clinical manifestations of EGJ cancer.

## Conclusions

Adenocarcinoma of esophagogastric junction rarely presents EGFR mutation, especially gefitinib-associated mutations such as L858R, or delE746-A750. This means that currently the gefitinib-based gene target therapy should not be recommended for treating the carcinoma of esophagogastric junction.

## List of abbreviations

EGFR: epidermal growth factor receptor; FDA: Food and Drug Administration SNP: single nucleotide polymorphism; NSCLC: non-small cell lung cancer; EGJ: esophagogastric junction; ESCC: esophageal squamous cell carcinoma.

## Competing interests

The authors declare that they have no competing interests.

## Authors' contributions

WWP designed partial molecular experiments, and drafted the manuscript. WKN and GQ performed partial molecular experiments and revised the manuscript. CLQ participated in the overall design, study coordination and finalized the draft of the manuscript. All authors read and approved the final manuscript.
